# Facing neoliberalism through dialogic spaces as sites of hope in science education: experiences of two self-organised communities

**DOI:** 10.1007/s11422-021-10042-y

**Published:** 2021-07-01

**Authors:** Betzabé Torres-Olave, Paulina Bravo González

**Affiliations:** 1 University of Bristol, Bristol, UK; 2 Pontificia Universidad Católica de Valparaíso, Valparaíso, Chile

**Keywords:** Democratising dialogue, Neoliberalism, Hope, Communities, Science teachers

## Abstract

In this paper, we discuss the role of dialogue in two layers; first, in relation to two self-organised communities of science teachers in which we participated and, second, our process of coming together during our PhDs to analyse these communities, a dialogue about the dialogue. Regarding the first layer, there is much to learn from science teachers and science teacher educators when they are organised in sites of learning that can be spaces of hope, beginnings, and becoming, as is illustrated in the case of these two self-organised communities. Regarding the second layer, we discuss the value of dialogue and the possibilities it offers to develop ideas for science education in a way that might be democratising, emancipatory, and offering counter-narratives in a neoliberal Chile. By engaging in this dialogue revisiting the practices of our communities, we gained a sense of agency within the field of science education. However, we realised that we need to move towards a critical view within our communities, and more contextual and transformative science education by translating these sites of hope to our educational praxis today. For us, this relates both to developing a collective view of how to make science education provide pedagogical conditions and experiences for critical and engaged citizenship and thinking how we can act and engage with different settings in solidarity. One way of moving towards this is by developing a political knowledge of our disciplines through a collective scientific conscientisation. Our communities are the departure points to achieve this.

## Resumen

En este artículo, discutimos el papel del diálogo en dos capas; primero, en relación a dos comunidades autoorganizadas de profesoras y profesores de ciencias en las que participamos y, segundo, nuestro proceso de reencontrarnos durante el doctorado para analizar estas comunidades, un diálogo sobre el diálogo. En relación con la primera capa, en nuestra experiencia hay muchas cosas que podemos aprender de profesoras y profesores de ciencia y de personas que trabajan en educación científica, cuando están organizadas en comunidades, como las dos que presentaremos aquí, como sitios de aprendizaje que pueden ser espacios de esperanza, inicio y creación. En cuanto a la segunda capa, discutimos el valor del diálogo y las posibilidades que ofrece para desarrollar ideas para la educación científica de una manera que pueda ser democratizadora, emancipadora y ofreciendo contra-narrativas en un Chile neoliberal. Al participar en este diálogo revisando las prácticas de nuestras comunidades, hemos ganado un sentido de agencia dentro del campo de la educación científica. Sin embargo, nos dimos cuenta de que necesitamos avanzar hacia una visión crítica dentro de nuestras comunidades, y una educación científica más contextual y transformadora al traducir estos sitios de esperanza a nuestra praxis educativa actual. Para nosotras, esto se relaciona tanto con el desarrollo de una visión colectiva de cómo hacer que la educación científica brinde condiciones y experiencias pedagógicas para una ciudadanía crítica y comprometida como con pensar cómo podemos actuar e involucrarnos en diferentes entornos de manera solidaria. Una forma de avanzar hacia esto es desarrollando un conocimiento político de nuestras disciplinas a través de una concientización científica colectiva. Nuestras comunidades son un punto de partida para lograrlo.

In this paper, we begin by situating our argument within current socio-scientific and political challenges. Secondly, we briefly discuss our understanding of dialogue and what it offers as a democratic practice. Thirdly, through our dialogue, we present two cases of science teachers’ communities, by reconstructing some of their practices as sites of hope. Then, by analysing the abovementioned dialogue, we create themes, (1) collaboration and trust, (2) recognition and value of what everyone brings, and (3) the urge to disseminate what they do. Finally, we argue that even though we understand our communities as sites of hope; we need to push our reflections forward, engaging in a critical type of dialogue towards both political content knowledge and a collective scientific conscientisation.

## Are there spaces of hope in a neoliberal context?

The world today faces many crises, such as climate change and the coronavirus pandemic, thus it is our duty to emphasise the importance of spaces of dialogue about love, hope, and collective care in science education. Even though neoliberalism strongly pressures us to enter the arena of competition, meritocracy, and individualism, we must resist by thinking with the other (Freire 1970) and by caring about the other (hooks 1994). In these spaces of dialogue which we introduce, we endeavour to resist this hegemonic neoliberalism and attempt to rehumanise science education.

Dominant neoliberal logic squeezes abilities to act and think collectively out of us, through the production of non-collective subjectivities (Rodríguez 2019). The rationalities and activities valued within neoliberalism have shaped the nature of education, along with educational goals and policies. As such, neoliberal logic also tries to erase from our imagination the notion of a different future and therein lies its triumph (Klein 2017). Nowadays, we struggle with ideas of competition, and inequalities implicit in the banking model, whereby the educator deposits information in learners, and learners are not considered active actors in the process (Freire 1970). This banking model is no longer used solely in education but has also become one of the most dominant forms of relationship between individuals and with the natural world. The same has happened in some traditions of educational research where an extractivist mode of interaction views experience as something to be mined and pulled out from others in order to create knowledge in a non-dialogic way (Santos et al. 2007). This logic creates a false dichotomy between knowledge “producers” and “consumers”, or those who know and those who do not (Camillo 2019). This dichotomy perpetuates a vertical model of relationships together with the hierarchisation of knowledge and subjects. We see this hierarchy reproduced in educational policymaking, teachers’ professional development programmes, pre-service teachers’ education, and schools. This banking and extractivist approach reinforces the role of universities as ‘ivory tower’ (Geduld, Baatjes, and Sathorar 2020) looming above the community, and allowing only some subjects within that community to be ‘knowledgeable’ about their own experiences (Popielarz 2020).

As example of vertical relationship, some of the repeated aims of science education include development of argumentation, collaboration between peers, and scientific literacy. The development of these aims is only required of the students and ignores entirely the people responsible for education—academics, teacher educators, and schoolteachers—should also possess those skills or attitudes (Bencze and Hodson 1999). Transformation should not only be expected from others, but also from ourselves. Moreover, the understanding of those aspects of science and education is mostly taken from western traditions that are presented as universal, ignore ontological diversity, and fail to consider what we can learn or create within our own contexts.

Scientific literacy—or scientific conscientisation as we prefer to call it—and especially the approaches used to achieve it, must be contextually driven and place-based (Roth and Calabrese-Barton 2004), not only because socially relevant science is important to people’s lives (such as in the case of the Covid-19 vaccines and various molecular diagnostic tests), but also because this place-based education will help people to develop an identity within knowledge and its production. As per Lyn Carter (2005): ‘science education agendas should emanate from local problems instead of from ‘global economic restructuring and the imperatives of the supranational institutions that are largely beyond the control of science education’ (p. 561). If we acknowledge the role of context, we might be able to move away from a banking logic and instead start from the grassroots and from within ourselves. This statement does not mean that we have decided to block ourselves off from trying to learn from other realities, but rather that we need a space where locality is discussed. To pursue a better understanding of science educators’ roles will not solve inequalities nor will it change the neoliberal model, but it will acknowledge and deepen the belief that science education can influence the creation of a future rooted in social and environmental justice (Bazzul and Siry 2019).

Thus, the vertical model influences many different areas of our lives. An example in the Latin-American context is the way in which the Chilean state deals with environmental crises such as the lack of water availability due to the privatisation of the distribution system, and the prosecution of those who denounce it (OCMAL 2017). Chile is one of the few countries that has a water distribution system privatised in its constitution (Mundaca 2014). When the depletion of water results in desertification, the private sector as a whole is not held responsible: with the influence of neoliberal logic, desertification is viewed as the irresponsible actions of a few individuals. Despite this, the neoliberal model, which so values privatisation, remains untouched (Kremerman 2019). All these make Chile a country beset by stark inequalities. In science education, these issues are often overlooked in the obsessive search for outcomes and products, making its political dimension absent or hidden, becoming the elephant in the room (Carter 2014).

As science educators, we should not rest on assumptions that stakeholders or politicians will solve the problem. We must act by changing our practice, and ourselves, through asserting that there is no neutral, universal, or objective ground for researchers and their work. Science education often works to preserve an internal agenda rather than take on a commitment to collective social transformation (Bazzul and Tolbert 2019). Looking outside science education is necessary in order to offer a rich view of the world as well as to learn from historical, political, and other narratives (Bazzul 2020). Such a challenge is especially important in the context of Latin America, where there are deeply set inequalities.

As newcomers in the western science education sphere, this lack of criticality in our context can appear as the duty of someone else. Should science education have social justice as one of its aims, rather than simply being focussed on instrumental, employment-orientated training of students? Not today, apparently, if certain opinions within neoliberal educational trends are to be believed. Thus, in some places, the hegemonic tradition reinforces the banking model that Freire critiqued so many years ago.

Despite these struggles and constraints, we trust that there are other ways to be a science educator in a neoliberal context (and beyond). For many of us, social justice is one of the reasons why we teach and engage with science education (Moura, Torres Jager, and Guerra 2020). An example unfolded during the ongoing Covid-19 pandemic when we started to write this article. We could see lights and shadows of scientific work. Efforts to produce a vaccine massively accelerated, vaccine development, and some groups of scientists were making data freely available to other groups around the world, depositing pre-publication manuscripts on sites like BioRxiv.org, rather than closely guarding their data and research as they usually might. This initial collaborative spirit from academics deriving funding mostly from public grants allowed a process that usually takes 10 years to take less than a year (with the Pfizer, Moderna, and AstraZeneca vaccines all now approved). On 13 April 2020, the webpage for the WHO reported that ‘Under WHO’s coordination, a group of experts with diverse backgrounds are working towards the development of vaccines against COVID-19’. This group of researchers made the following declaration (extract):

We are scientists, physicians, funders, and manufacturers who have come together as part of an international collaboration, coordinated by the World Health Organization (WHO), to help speed the availability of a vaccine against COVID-19. While a vaccine for general use takes time to develop, a vaccine may ultimately be instrumental in controlling this worldwide pandemic. (WHO 2020)

In another paragraph in this declaration, we were struck by the reference to ‘worldwide collaboration, cooperation and sharing’ and the word ‘unprecedented’; this collaborative effort to address disease should be one that occurs normally and not only in a global crisis. Even though this effort calls for global collaboration, it is important to examine what is happening in each local context to consider how diseases impact each separate community, such as the availability of hospital beds or access to medicines. Thus, a situated view examining the local context is necessary. Although this ‘unprecedented collaboration’ gave us some hope, at the same time, we knew the pharmaceutical industry will want to extract profit from vaccines produced in certain countries, while health continues be seen as a luxury instead of a human right, which will ‘first’benefit those nations with more power. This has subsequently become the reality, as vaccine candidates have moved from the sphere of academic to commercial development. What was initially publicly funded, and publicly available research data have been taken and transformed into potential vaccine candidates behind closed doors by the for-profit pharmaceutical sector, followed by arguments ensuing about first distribution rights. Unfortunately, science has also had responsibility in generating an entrepreneurial mindset in issues of health care such as pharmaceuticals market (Weinstein 2016). Ethical positions for a collective good may help to face these issues of power. Depoliticising science education does not help to challenge these views.

However, we still maintain the politics of hope, which involves ‘the art of using knowledge creatively and politically to weave dreams out of misery, against the odds, amidst brutal state violence, endemic poverty, desperate hunger and social devastation’ (Dinerstein 2015 p. 26). How can we achieve this in educational settings? For us, when science teachers working in schools and science educators working at the university context are able to discuss their daily practice together, a site of hope is created (Torres-Olave 2020). That space itself is an important part of the interaction. In our experience, the self-organised communities are creating those sites of hope.

Coming back to the initial question of “are there spaces for hope in a neoliberal context?” We say yes! To build upon that affirmation, we would like to offer our experience in the self-organised communities we are part of, where dialogue has become a crucial mode of interaction related to this desired hope.

[h]ope as a form of oppositional utopianism is one of the preconditions for individual and social struggle and the ongoing practice of critical education in a wide variety of sites—the attempt to make a difference by being able to imagine otherwise in order to act in other ways. (Giroux 2017, p. 121)

That is our commitment.

## What do we mean by dialogue?

Dialogue can be characterised as the act of talking about an issue that has particular purposes (Lefstein and Snell 2014). It can also be conceptualised as a social and political issue that involves deeper interpretations of communication and social relations, such as power and cultural tradition (Burbules and Bruce 2001).

The dialogical relationship will depend on what participants are saying, where they meet, how they position themselves, the gestures they use in the interaction, and any other activity or work involved. In this sense, the dialogue as a discursive practice has materiality which is worth paying attention to when trying to understand circumstances in which it occurs (Harste, Short and Burke 1988). Opposed to this materiality, there is a notion of idealised dialogue, organised around norms of egalitarian, open-ended, and reciprocal communicative interaction (Lefstein 2010) which is contrasted, to name one example, to the imperatives with which the curriculum has been created in Chile, and the historical struggle between teachers and curriculum developers (Ogborn 2002). In Chile, teachers are mostly considered curriculum implementers (González 2015). A recent example of this is the incorporation of the big ideas’ framework to the Chilean science curriculum. In 2012, a Spanish version of the big ideas report was launched, and that same year the big ideas’ framework was incorporated into levels 1–6 of schooling. In 2015, it was expanded to levels 7 through 10, and in 2019, it was included in levels 11 and 12. By 2019, the approach was therefore incorporated into every level of schooling except pre-primary education. The incorporation of the big ideas was not discussed by the schools nor teachers or students of the Chilean educational system. Rather, it was introduced by the Ministry assuming a world tendency. This assumption was expressed as “According to the world’s trend” (MINEDUC 2015, p. 18) or the review of the “International comparative experience” (MINEDUC 2019, p.11), disclosing that the incorporation in the curriculum was achieved without teacher dialogue.

Considering the aforementioned definition of dialogue, it is possible to identify different dimensions of dialogue. Firstly, the ideational dimension, where dialogue is valuable for its potential role in developing cognitive abilities and conceptual tools, whilst also adding creation of knowledge. In the same vein, dialogue can be constructed as a fusion of horizons as pointed out by Gadamer (1998), where engaging in dialogue with others is an epistemological position that allows a self-understanding and a revision of our own horizons. Similarly, Freire (2005) pointed out an empowering understanding of dialogue when the social dimension is included as a way of knowing. However, as pointed out by Lefstein (2010) one element of dialogue as communicative activity is the acknowledgement of tensions. Instead of disregarding the tensions that, for example, the same dialogic movement can generate—e.g. students who do not want to engage in an ‘empowering’ dialogue—addressing them may reduce idealistic forms of dialogue as egalitarian per se. In that example, dialogue might be experienced as repressive. Ellsworth (1989) asked the sensible question of “Why doesn’t this feel empowering?” (p. 317) acknowledging that dialogue involves also the exercise of power. Whose knowledge is more legitimate? Who has the authority? Which identities are privileged? These types of questions are the ones we asked ourselves, and to the self-organised communities regarding dialogue, trying to be aware of the tensions of uneven power relations which is explored later in this document. Ellsworth offered an answer to these conundrums suggesting that “the only acceptable motivation for following others into their worlds is friendship” (p. 317) depicting the importance of the interpersonal dimension of dialogue. An essential interpersonal dimension of dialogue is listening to the other, as an act of recognition (hooks 1994). Dialogue should encounter and develop emotions such as trust and affection (Burbules 1993). We accept the other’s point of view, and in so doing, we build with them, rather than defeat them (Burbules and Bruce 2001).

Considering the ideational, epistemological, and relational dimensions of dialogue, Paulo Freire (1970) evokes our questioning by asking how can we dialogue if there is a power imbalance that is favourable to the elite, and the concurrent displacement of others? For us, the neoliberal context generates what Freire described as a non-dialogue/monologue way of interacting where recognition is just for a few.

### How can we face the power imbalance that generates a non-dialogic culture?

We attempt to answer this question by presenting the self-organised communities that we are part of. In doing so, we propose to see dialogue in two layers; first, in relation to the way, we experience dialogue inside the communities of science teachers and teacher educators (educators of educators) and, second, our own process of coming together during our PhD journey to analyse those communities, through dialogue about the dialogue.

In our process of becoming researchers in science education, we wish to create insights and shed light on these teachers’ communities that have taught us so much and have been shaping us for so long. Currently, distancing has allowed us to ‘observe closely’ and learn, once again, with them. This is what Freire (1970) calls ‘epistemological distance’, renaming the world (Steinberg and Kincheloe 2012) and being able to see it from another referential system. In this dialogue, through a process of conscientisation (Freire 1970), we might be able to question our notions of science, learning, education, scientific literacy, and theoretical thinking.

Through sharing our experiences in becoming researchers, we have found common points like our interests in dialogue through dialogue. Maybe that was because we missed the safe spaces that were previously provided by the self-organised communities, and in our being so far away has in some sense made us come closer. We both studied for our undergraduate degree in the same city in Chile where these communities began. In this city, we were not exempt from the principles of individualism in everyday life. However, there were also these communities where we lived in collaboration. This might sound incoherent since we said that neoliberalism permeates every aspect of our live. However, we believe that in Latin America we also have this sense of collectivism which is captured in Gioconda Belli’s (1981) phrase that ‘solidarity is our tenderness’ (p. 157). We live in a land of contradictions where the neoliberal model shapes us, but we are resilient, agentic, and with a collective spirit inside us which is probably also related to this Freirean idea that we cannot be without another. In these communities, we learnt how to be within the world of science education, and then how to be in relation to others. Now, we look for others to share our learning and experience because we cannot conduct research or writing as an isolated process. This is because we understand it as a cogenerate process, always in dialogue with someone or something. In so doing, we started this conversation months ago, by sending each other messages, emails, pieces of writing, visiting each other, sharing our readings, our doubts, and mostly learning from each other in constant questioning that we learnt from before, and we explain below.

How can research in science education help transform and overcome environmental and social inequalities? In what ways can science education research contribute to improve our professional praxis? In what ways can research in science education embrace the idea of community as an inclusive learning enterprise? We consider these questions as a means for opening a new standpoint to rethink science education research. Herein, we would like to share two exemplars of collaborative dialogic spaces in the city of Valparaiso that we depict as spaces of hope, illuminating our process of becoming researchers and facilitating dialogue along the lines discussed above.

In the following section, we alter the structure of the article. From now on, we will be writing as ‘Betzabé’ and ‘Paulina’ to show you our own dialogue (face to face and written by email exchange) introducing the self-organised communities of science teachers.

## Locating through our dialogue: the self-organised communities

*Human beings are not built in silence, but in word, in work, and in action-reflection.* (Freire 2005, p. 88)

Betzabé: I think we should start with PRETeC [Profesores Reflexionando por una Educación Transformadora en Ciencias; Teachers Reflecting for a Transformational Science Education in English] since it is the oldest self-organised community in which we have participated; so, you should talk about that. But first, should we talk about why we are saying self-organising community?

Paulina: Brilliant, since you were the one offering the term, why don't you explain the concept?

Betzabé: I think we should call our communities ‘self-organised communities’ because they are born from the initiative of teachers and teacher educators, and not from an institutionalised logic where someone tells you that you need to get organised. It is an invitation not a mandate. These communities are the result of teacher’s agency as a first move towards something bigger, and I feel the framework of the communities of practice may not capture that agentic essence. In a way, these communities are examples of democratic professionalism, or what Stephen Kemmis (1993) calls ‘critical communities’ so I would not call them communities of practice. Although they share internal characteristics of a community of practice, there is something more here. Critical communities, in Kemmis’s idea, have reflection, self-knowledge, and are action-oriented to transform the educational practices that facilitate possible changes to make society fairer, more democratic, and humane. Maybe there is still some way to go before they become all of that, however, as we said, these are spaces of hope and this description also captures that. These communities were born in a country that has a history of constrained teacher’s organisation so the use of the free time to work with others outside your workplace is something that one decided for oneself, then that becomes a collective commitment. That is why, I think, we should call them self-organised communities, to embrace that sense of agency within the name and to leave that room for more and more actions. Maybe a self-organised community is a type or community of practice (Wenger 1998), but I think we should use this nomenclature to emphasise that are not communities within the same school, or a formal partnership led by a one-time research programme. So now, you can introduce us to PRETeC since you are one of the founders of this community.

Paulina: PRETeC started around the 18th of June 2010, when a group of teacher educators from a university in Valparaíso made an open call to a group of schoolteachers to be part of a nationally funded research project. On the first day, 16 schoolteachers arrived, six from a previous research project and 10 new teachers. The initial event was tempestuous because the new teachers did not trust these three women—Corina, Mónica and me—who were inviting them. They questioned our experience working in a school—which in fact we did not have—and the real impact that the project could have in their workplace. Now I understand that this concern was totally right, those teachers were accustomed to receive this kind of training or being part of projects where opinions, experiences, conceptualisations and so on were handed down, the banking logic, that is to say, the neoliberal way to interact as we stated at the beginning of this article. As I said, now I understand, but at that moment with my colleagues, we were horrified, and we felt that our journey began as a failure. Even though this first encounter was difficult, from the second and the subsequent meetings the same group of schoolteachers have been coming back every Friday between 5 and 8 pm-ish for almost 10 years now. Putting myself forward as the advocate of the group, the aim of PRETeC has been to share our practice in science education, discussing some aspects such as the image of the science teacher, the importance of science in the life of our students, and in our own lives developing our own conceptualisation of science education in Chile. The number of participants in PRETeC has changed, now the group is made up usually of more than 10 people from different backgrounds, stories, lives, etc. but having in common this urge to meet and talk about what we do regarding science education. The way we share our practices inside the group since 2013 has been through something we called lesson stories, which are the written descriptions of the activities that every teacher carries out in their classrooms adding both reflections and obtained results; nevertheless, the way we share the group’s work is through some publications and a book—I cannot help talking about the book. It is the document we are most proud of, because we wrote it collaboratively over 7 years (PRETeC 2018). Coming back to the lesson stories, Betzabé do you remember that once you presented one lesson story?

Betzabé: Yes, that was when I finished my Masters in Bristol and I went back to Chile for three months. I think participating in those meetings for that short amount of time was key in changing my research topic from the teaching of the nature of science to science teachers’ agency within these two communities. I think that experience of sharing your lesson in a written manner, it's like a meta reflection because you cannot change what you wrote so you read it. While reading it you are reflecting on it at the same time others are actively listening to it, then we discuss and problematise it. How did you come out with that form?

Paulina: The lesson stories started in 2013 after the visit of Ian Mitchell^1^ who showed us the way he and a group of teachers in Australia share their work. They called it ‘cases’ and we took that form and translated it to our reality. The mode of working around these lesson stories^2^ is by reading the written accounts by the author and then we discuss during the session. For example, a lesson story shared in one session was related to the consensus that Pluto was no longer a planet and the students did not understand why that decision was taken; in that session, the group reflected on the nature of science and gave ideas to the lesson story’s author to keep developing that understanding of how the knowledge is built. One idea was to develop with the students a biographic review of the people involved in the classification of the planets, to understand where, how and who were those people.

Betzabé: I like that you said, “we translated it”, because you did not take the idea as it is. You embrace the idea and contextualise it and one thing that I really like about it is that is not just “my best lesson”. I was part of some sessions where people were really struggling with some issues in their lessons and it was amazing how everyone engaged with the story, rescuing ideas and offering new paths as well. The start of the physics teacher’s community was a bit different. I do not know if you remember once back in 2010 when we had a meeting at the University, and you recorded it.

Paulina: Yes, I remember it! I was there just to ask some questions and gather information. I also remember that we were not as close as we are now. We were working together on some projects but now we are friend-friends who are more productive because, as Freire would say, friendship has become a generative theme. Before diving into those aspects, please, go ahead presenting the self-organised community.

Betzabé: I like that idea of our friendship as a generative theme. Indeed, this dialogue is the product of this process of coming together. A generative theme is a concept anchored in reality in Freire’s work, and our friendship is that both in the academic and non-academic world. When I talk to you about our research or any other thing, I realise new ideas, and also concrete possibilities to my practice. Regarding our community, this recording I was mentioning was made just under an exploratory logic of doing research from a naive approach, trying to imitate what the maths teachers were doing at a local secondary school to encourage reflection on practice. Some of us were still pre-service teachers at that time. After that, the same group of people were once in someone’s house and some of us were struggling with teaching some content, mainly because teachers do not usually have an induction in the schools where we work and also because it is not common to have more than one physics teacher within a school. We were new teachers, so it is also normal to realise that you do not know so many things. So, we decided to plan some content together and we started with forces and movement. That year, I think it was 2012, we did this water-rocket activity—this has been a yearly activity since then with different versions—but in our own schools and sharing some previous ideas for introducing the concepts, the design, and so on. The year after, other teachers (that we knew from the University) decided to join us and someone came up with the idea to do this activity every year using different places in the city since we live in a seaside city with a really nice landscape. We did it, and it was great. We put together students from public, semi-private and private schools in a beach in Valparaíso, and that was the first time, for me at least, that teachers from other subjects in the school were really amused with physics and that there were possibilities of doing collaborative work and engaging students with physics phenomena. This activity then became the joint activity of a group of nine physics teachers that now meet regularly because it is not just this event that happens every year now for 5 years, it is a lot of other things as well. It is about science communication, public engagement, collaboration with pre-service teachers that now also participate in the activity, and all the meetings they have before where they talk about more than the activity itself. They also discussed and problematised how to teach this content at different levels, some of their problems while teaching, the assessment and then learning from some positive and negative experiences and even the possibility of inviting people from other cities to collaborate. That is the level this collaboration has taken. They now also work making students from different schools mix with each other solving some problems regarding physics concepts that are part of the analysis of the water-rockets. Some teachers in the group are also organising other types of encounters, concerning other subjects within physics, like robotics.

One thing that I would like to call to attention is the value of each other’s experiences that are brought to the meetings and are shared between everyone, even when some of them are not teaching at the school anymore. This shows that they all have something to offer to this space. Besides, they declare that it is a way of refreshing themselves, to achieve real collaboration, and also as a way of improving their own wellbeing. In a group dialogue last year (in the context of my PhD thesis), some of them said that they would like more people to know what they do, not expecting it to be replicated exactly as it is, but maybe enlightening new ideas of collaboration using the local territory as a living laboratory. Or maybe, as you did as well, translating this experience to other contexts. Others also say that they feel they know more about pedagogy and the curriculum thanks to this experience. This was because they put their assumptions into dialogue with others, in a trusting environment, where it is not a problem to present an unsuccessful experience. In addition, for some reason, they have more ideas also to use in the school where they work, thus translating this new knowledge from one place to another. The process of collaboration is transformative since it goes against the traditional lone working and encourages reflection that is then translated into actions.

## Analysis: going inside the communities through the lens of dialogue

Now that we have presented these self-organised communities, we will present our dialogue about the preceding dialogue. The method by which this was achieved involved an initial face to face conversation about what we knew and said about these communities, organised into themes:

- Collaboration and trust,
- Recognition and value of what everyone brings, and
- The urge to spread what they do.

### Collaboration and trust

Participants commitment to collaboration was a common theme identified in both communities. Under the neoliberal logic of the Chilean educational system, this is odd, at least because these spaces do not exist in their conception of the education system; on the contrary, it presents some constraints to work in this way. For example, a space that should be collaborative is the “Consejo de Profesores” [faculty council] which should present an opportunity to talk about pedagogical issues, as well as particularities of the profession and praxis within the school, with an agenda that is decided by the teachers themselves. However, this space again takes the neoliberal logic of verticality and non-dialogue. In an ethnographic study carried out over three years in different schools in Chile, Felipe Acuña, Paulina Contreras, and Jenny Assaél (2019) found that a common feature of these spaces was the vertical logic and silence. This culture of silence is not something new in the Latin-American context, for example, Freire in *Pedagogy of the Oppressed* (1970), and in *The politics of Education* (1985) pointed out how the culture of silence, in opposition to a process of liberation, gained ground under the context of the (often decades long) dictatorships established across Latin America during the cold war, which has been perpetuated until today with the processes of privatisation and marketisation under the neoliberal an economic trajectory begun under those dictatorships.

The culture of silence has been a key element in the system of oppression which became a hegemonic tool of the elite forces (Gramsci 1974) making it the ‘common sense’ discourse. Understanding the imposition of silence as common sense allows us to understand certain characteristics of our culture today (Baeza 2019). The culture of silence was reinforced in such a way during the Chilean dictatorship so people grew up in an environment where there were issues about which they could not discuss openly, such as disappearances, and torture. This culture of silence permeated life in the school where authoritarian practices were normal, since those who oversaw the schools were from the military, in some cases civilians appointed by the dictatorship, in other cases soldiers appointed to run schools. This has had a profound effect on the childhood and adolescence of today's adults, our parents’ generation, our colleagues, and our teachers. They were denied their subjectivity, and silence was imposed on them as the norm, with the voice of authority as the only valid truth (Baeza 2019).

We can still see the effects of this ‘common sense’ of silence in our educational biographies where the teaching style was usually carried out according to this banking model, and a good class was measured by the levels of noise, or lack thereof, without opportunities, as students, to put our beliefs into dialogue with this new information. The result of these teaching practices led us to believe in the universal truth of authority (hooks 1994). Despite Chile no longer being a dictatorship, the culture of silence seems as deeply rooted now as ever. It is not just the culture of silence but also a non-dialogic attitude, meaning refusing to talk between assumed hierarchical relationships (Acuña et al. 2019). Teachers feel that their voices are not valued by the school, while the school administrators are also refusing to talk, and in an environment that is not driven by trust. Mandatory requirements by the ministry together make a chain of non-dialogue across institutions. Together with the culture of silence, there is also now an apparent conscious decision to not talk in a place where one feels neither safe (hooks 1994) nor heard (Freire 2004).

The previous idea about the culture of silence aligns with what Carmen Montecinos and Mónica Cortez (2015) found regarding teachers’ communities creating dialogic spaces outside of the school context. If the community exists in a culture of non-trust, e.g. the school, or if collaboration and collegiality are imposed and not sustained in time, the community is not significant for the teachers. Furthermore, in the Chilean educational system, collaboration is a dimension of the national teachers’ assessment, wherein teachers are required to collaborate at least once per year with their colleagues. It should be illustrated that describing that particular experience is an integral part of the assessment portfolios, which determines many aspects of the teachers’ career, not least their pay the following year, which in Chile is not determined by collective bargaining of national pay scales, but rather is based on individual performance. In doing so, the conceptualisation of collaboration is reduced to an event that is assessed, which could be seen as artificial collaboration (Hargreaves 2003) or contrived collegiality (Hargreaves 1994), where it exists just to tick a box on a checklist. To furnish this argument with an anecdote, one of the teachers in these communities wrote of her experience in the community as an example of her work in collaboration across the year, particularly, the water-rocket inter-school activity organised with teachers across different schools in the city, which was not counted as collaboration by the assessors. This meant that she missed out on being awarded the ‘points’ for that part of the national assessment. In this sense, what would count as collaboration at the policy level in the Chilean educational system? Apparently, the answer would be what the national assessment wants to impose in the sense of what they consider to be appropriate teacher collaboration and excluding anything outside of their narrow parameters of control.

In the self-organised communities, the culture of common practice shifts. There is no longer a culture of silence. Conversely, there is a culture built around the ‘necessity of talking’, and an urgency to say the words that go in line with the very nature of the teaching profession: a dialogic dynamic. Teachers need collective spaces to create personal views and collective purpose, both things are part of the core of pedagogical praxis which is commonly missed in the school context (Cornejo 2012). The work of Carmen Montecinos and Mónica Cortez (2015) exploring the experiences of three self-organising teachers’ communities in Chile summarised three key issues that exist in these groups. First, the communities understand professional development and collaboration as a process, and not as an event that occurs in isolation as, for example, in the teacher assessment once every 2 years. Such is the case of PRETeC, who have been together since 2010 until the present day, and the self-organised community of physics teachers with almost 5 years of continuous operation. Second, there is the intrinsic motivation of wanting to do something better, rather than being driven by external incentives. The water-rocket activity is a yearly event out of the school, which encourages and aids the students to work around physics content, collaborative work between peers, and collective activity between schools. Third, professional learning amongst peers enables the development of another type of relationship which is called a ‘culture of trust’ by the authors. González-Weil et al. (2014) proposed some principles regarding how to conduct professional development between peers stating that they intend to reflect individually and collectively on the exercise of practice in an environment of respect, trust, and mutual support. It is this culture of trust that is an essential part of the fabric of what the group is and intends to be. Teachers feel more confident discussing issues about their practice between themselves as the relationship is non-hierarchical and collegial without accountability pressures. For science teachers, this culture of trust is particularly important since it is rare for more than two science teachers to work at the same school, and there is usually only one per school in the case of physics teachers. This leads to a situation producing isolation, and a situation where teachers lack spaces for collegial dialogue about subject knowledge, socio-scientific issues, or didactics of the discipline.

Another similar theme closely related to the culture of trust is the emotional factor involved in these communities. Teachers that are part of these communities created a sense of identity with each other. Developing an identity collectively is emotional since ‘it involves processes of becoming which are associated with visions of self, goals, aspirations, beliefs, and enculturation in specific social, historical, and geopolitical contexts’ (Avraamidou 2019, p. 337). In this collective becoming, teachers also exercise their agency since these communities originate from the teachers’ own initiative, they are responsible for their own development and learning, and capable of sharing and projecting their goals regarding science education. This exists in relation to others (Freire 1970), which brings us to the recognition and value of what everyone brings, which, in addition, is associated to the urge to spread what they do.

### Recognition and spread

As discussed earlier, dialogue has different dimensions. Dialogue is a way of creating knowledge through process of tensions within and between each other (Darder 2002) and that also serves as a way of revisiting our horizons (Gadamer 1998). However, in a culture of non-trust, engaging in that type of dialogue is not possible. To make dialogue happen, it is necessary to fulfil certain conditions. Time is needed for dialogue to flourish. The emotions of participants need to be appreciated, affections and hopes need to be made visible. (Burbules 1993). These emotions may not appear if we do not recognise that what others bring with them to the community is ‘as valuable as what “I” could bring’. Recognition is particularly relevant in the world of science that historically has marginalised some groups such as women (Avraamidou 2019). We as women working in science education have experienced that exclusion within the institutional culture of science. We do, however, feel recognised and valued in these communities as other participants do because there is a positive feeling to value the professional experience of everyone equally, teachers and teacher educators (PRETeC 2018). This recognition is in the sense of knowledge and ideas that people bring with them, as well as experiences they want to share and problematise. The logic in this sense is non-hierarchical, the other now exists and contributes to my own view of science and to my hopes, and the other is now eager to change as well as me (PRETeC 2018). Recognition also exists in the way that these communities attempt to overcome the vertical nature of traditional professional development programmes (PDP).

Most commonly, PDPs in Chile aim for schoolteacher actualisation on some curricular content, in that sense, teacher educators are the ones assumed to have the knowledge while the schoolteachers are assumed—implicitly or explicitly—to be lacking that specific knowledge (Bravo, unpublished). In these self-organised communities, the recognition in PDP works in a different way, for example, in the same article about the principles of how to conduct professional development, González-Weil et al. (2014) proposed principle four; which suggests valuing the authority of experience as a source of professional learning. This refers to the recognition that the experience of the teachers in their schools should have an authentic way of building knowledge about the process of teaching and learning. When asking what makes possible the continuity by recognition of the other in PRETeC as a self-organised community, there are two processes that Felipe Acuña and Paulina Bravo (2019) reported as relevant. The first relates to the way in which the group allows those who attend to experience a critical review of their professional practice with the aforementioned lesson stories in such a way that it is experienced as a dialogue between equals. Teacher and teacher educators both share their experiences, so they all have the opportunity to transform their practices after collective feedback. The second relates to the way in which the community took the group’s own challenge of describing themselves and their work in the form of written products such as the articles and the book both being co-constructed between teachers and teacher’ educators over years. Those processes signify the recognition of the experience of the other for meaning-making and collective learning at the same time as allowing the spread of what is done inside the self-organised community.

Finally, we can also see a sense of mutual commitment or university-school partnership. Both self-organised communities have been participants at national conferences in Science Education, sharing their experiences working together through the years. For them, it is important to share how being part of a self-organised community makes them rethink their educational practices as well as problematise their views on science education. In those presentations, schoolteachers and university teacher educators work together to share their experiences within the community in a manner that breaks the traditional vertical relation between researchers and teachers. Both schoolteachers and teacher educators are now researchers and learners. They are now creating new knowledge in dialogue (Freire 1970) and showing that it is possible to hold this way of working together over time and not just for a particular one-time project.

This partnership that is created by being part of a self-organised community creates ties between the local University and schools, creating a commitment on both parts. For example, in a city where is a shortage of physics teachers, the participants in the physics teacher community usually work as mentor teachers for pre-service teachers. This task means extra work since there are not formal guidelines or extra pay to be a mentor teacher. It also involves trust with university teacher educators and with future teachers that take an active role in teaching science. In the physics teacher’s community, they also invite pre-service physics teachers to their annual water-rocket activity. They do it because they value and recognise pre-service teachers’ ideas and knowledge about science. They also do it because they understand that the goal of teachers is to spread this sense of agency they feel while working in collaboration and using the local territory as a live laboratory, which may also impact pre-service physics teachers’ identities (Adams, Miele, and Powells 2016). Sharing these experiences illuminates more collaborative ways of being a science teacher. This is as it should be because science in itself is a collaborative and sharing enterprise, albeit sometimes its practice demonstrates the contrary. This process can also enlighten us in the way we are doing research, as these spaces allow us to think in a different way about educational processes, outside the institutional and prescriptive logic (Cornejo and Reyes 2008).

## Learning and hopes

*Without hope, there is no way we can even start thinking about education* (Freire 2007, p. 87)

One of the most significant aspects of our participation in these communities relates to our personal and professional learning. Participation in these spaces has moved us towards writing this and talking to each other in the process of our research. Thanks to these communities, we understand the value of identity and agency and the relation between them, and how collective agency means the development of collective identity and how identity, in its turn, encourages a sense of agency. We have learnt that knowledge can be created differently, in dialogue, in solidarity, with engagement and commitment. We also hope that we have learnt the need for continued growth within these spaces, attempting to move in the direction of social and environmental justice considering the scientific and socio-political context. Maybe for some this is not a new discovery, however, because of our own cultural context it represents a beautiful starting point for us, from where we could attempt to break this banking model of life challenging the current modes of domination (Shor and Freire 1987) by engaging with others through a democratising and emancipatory dialogue.

The dialogue we have been experiencing might have been changing ourselves in a way that could be answering the Freirean called to an ontological hope (Freire 1993). If we see and conceptualise these self-organised communities as dialogic spaces and spaces for hope as we think we see them, we should start acting today. Acting today means engaging in the political sphere that has been oblivious to the science education dimension, again, the elephant in the room (Carter 2005). For example, how can we translate into our context what it is to discuss socio-scientific issues? As an example, Chile is amongst the top ten countries with the most environmental issues in the world, and it has at least 37 environmental conflicts across the country (Quiroz 2019) which could serve as scenarios to discuss in science education. Socio-scientific issues are political, and we need to make that explicit. We need to go beyond local experience since these two communities that we are part of are located in the same city. Recently, a teacher’s organisation in Chile made a clear call to everyone working in education with an emphasis on teachers. The call said that teachers should take individual and collective control of their pedagogical practice, through their words, experiences, and transformative actions. In doing so, teachers can move from revindication towards a transformative movement (Reyes and Gonzalez 2019), from denouncing to announcing and changing. We should understand this call not just for schoolteachers, but also for teacher educators and researchers.

What do we think this means for these self-organised communities? Our interpretation is that we need to move forward to a critical dialogue. We need to continue in the same vein we have been working in the self-organised communities. In addition, we think that we can introduce a concept that we call ‘Political Content knowledge’ (another PCK) similar to that proposed by Lee Shulman (1986) regarding the pedagogical content knowledge (original PCK), but focussing on the political aspects of teaching at school and university level. This means involving ourselves and the students in discussing controversies linked to the field of science in our territories and classrooms from a micro and macrolevel. For example, encouraging sustainable actions or discussion into how our actions affect the world, but without losing the attention to how industrial activities, impact and affect nature and our way of living (Quiroz 2019). An example of this could be the environmental problems in Puchuncaví—a borough in the region of Valparaíso—due to the presence of a hydroelectric dam and a copper mining in the region (Garretón, Somma, and Campos 2018). This means engaging with social action, facilitating students’ critical agency, and releasing ourselves from the fear of losing control (hooks 1994), for example, going out of the school to see the damaged territory and creating student-led research addressing those issues. We are conscious that knowing about what is happening is not a direct solution. However, it creates conditions for students’ production of knowledge about the problem (Morales-Doyle 2017). We know that this task is difficult because of the many constraints in the science curriculum and the neoliberal context, which is full of accountability pressures within teachers work. However, the critical democratising of dialogue helps us to see possibilities where conscientisation is not just raising awareness but taking action against the grain. Moreover, we think engaging with socio-scientific issues in a critical way should also be an essential part of science teacher education. The same activities we are proposing for schoolteachers can be done by teacher educators at the university. For example, systematic walks around the city to identify environmental problems that affect the communities the university is part of (Geduld, Baatjes, and Sathorar 2020), seeing how this could be address or problematise through the science curriculum for their future practice as teachers and their current actions as citizens. Outdoor science experiences encourage not only learning about the relationship between nature and society (Dillon, Morris, O’Donnell, Reid, Rickinson, and Scott 2005), but also context, community needs, and social aspects which are linked to the political dimension and distribution of power and wealth (Hodson 2003).

This move towards the development of a scientific conscientisation may not be as difficult as we might think. Students engaging with the political dimension of education is not new in Chile (Donoso and Somma 2019). The Chilean student’s movement in 2006, 2011, 2019 has been key to catalysing the change in the law in the education and political system (Rodríguez 2020). Thus, we recognise them as active key agents in social transformation. That is also our duty, to engage more with schools, with universities, and teacher education programmes, to strengthen the bridge between the university, the school, and its communities, and to cross it, meaning teacher educators working closely with schools and opening the university, connecting it with the life of the communities it belongs. Counter-hegemonic spaces, with new meanings and new practices, can help to plan and develop these actions (Burke and Bazzul 2016). We argue that these self-organised communities could come to resemble these counter-hegemonic spaces. In doing so, the political content knowledge could serve as the opportunity to also develop the political-pedagogical plan that Freire was referring to in Pedagogy of the Oppressed, which allows us not just to keep thinking of the importance of teaching science and the praxis within, but also what we have been developing in our PhDs related to a political dimension of science education.

We intend to make use of the richness that socio-political studies in science education can offer to the current situation in Chile, especially an approach in research that makes democratic dialogue with others the base for learning and transformation. We need to value the particularities that are offered by understanding the situational nature of dialogue instead of obsessing about generalisations. When we talk about change, we usually do so in relation to others: we talk about teachers, about students, but what about us? The way we learn, the way in which we understand and create knowledge? That is also our learning with our communities, and our call to reflect on the previous questions and learn with others where everyone is collaboratively seeking a change by respecting what is meaningful to the group and the individuals. Meaningfulness varies amongst participants and is inevitably locally and relationally contextualised.

## The road ahead

One way to address the social and cultural relevance of the curriculum and face neoliberalism through sites of hope is to guide the goal of science education for social and environmental justice and take an activist stance towards this. This is not based solely on analysing how science has impacted or how it affects the social world, but instead focuses on changing the daily practice of doing science education (Calabrese-Barton and Tan 2010). One example that came to us while writing this article was the news about 48 groups of (male) scientists in a competition for the creation of the first mechanical ventilator made in Chile (La Tercera 2020). One aspect that was called to our attention was that we had 48 groups of scientists using their labour in a competition, instead of collaborating to achieve something that is for the common good. After a couple of months, the participating groups decided amongst themselves that the objective was best served by forming an interdisciplinary collaboration instead of competing with each other. This might be an example of how to work with a scientific political content knowledge mindset, and problematise with our colleagues and students about it. Problematising how the culture of science became a competitive enterprise, the reasons behind it, but also how to act differently by creating counter-narratives. Politicisation of ‘science education can be achieved by giving students the opportunity to confront real-world issues that have a scientific, technological or environmental dimension (Hodson 2003, p. 654). However, in doing so, we as teachers and teacher educators need to see this first and stop denying the political dimension of science. Our self-organised communities are a place to start working in that line. For example, collectively developing our scientific conscientisation by studying and problematising the science curriculum to identify potential content that can be explored in our lessons considering its socio-political dimension and linking it to critical socio-scientific problems in our territories. In this way, we could first frame socio-scientific problems together (Levinson 2010) and create collaborative pedagogical materials that can be used both at school and the university with pre-service science teachers. Some examples in our context could be discussing issues such as unequal access to technologies which has been made evident with the current pandemic, or particular towns where environmental crises affect the lives of people (dos Santos 2009), or as we mentioned at the beginning, the privatisation of water. There are many things to explore that both us and our students experience in our daily lives.

Chilean students have taught us how powerful they are and have demonstrated their ability to catalyse change. For example, the recent protests that erupted in Chile in 2019 where initiated by student protests against a fare increase on the Santiago metro system (Somma, Bargste, Disi Pavlic, and Medel 2020). This protest quickly escalated into a nationwide movement demanding constitutional reform. It is the time for us to provide them with more tools to continue to contribute to the national political debate, while also learning with them. Enactment of the science curriculum could incorporate critical social problems in the lives of our territories when they intersect with natural, scientific, or technological phenomena.

This should not only mean rethinking the science curriculum but also understanding it as a structure which should be discussed critically by the people involved (Morales-Doyle, Varelas, Segura, and Bernal-Munera 2021). Jesse Bazzul, Larry Bencze and Steven Alsop (2019) point out that ‘as educators we need to begin acting like solidarity is one of the default ontological/axiological positions of our shared world(s)’ (p.i), in this line, we have gained ground since we believe solidarity is culturally engrained into the fabric of Latin America. However, we need to advance in our communities and stop just reading and explaining reality. We need to enunciate new ways of relating to each other (Freire 2012). How do we create science education pedagogies that are connected and engaged with students’ lives that struggle to overcome the dynamics of the banking model? This task is not only for schoolteachers, but also for teachers’ educators as we work in the education of future teachers. By creating and being part of these communities as places of dialogue, we have already taken some steps along the road since we have a place where we can put these issues to discussion. Nowadays, we need these sites of hope and collectivism as cracks of lights within neoliberal societies.

In summary, we live in Latin America, a territory of inequalities. However, we can also see hopes that need to be translated from ‘scientific’ issues to public concerns, not just between us but also to spread through our educational praxis across settings and in our way of life. Teachers and teacher educators’ engagement should go beyond our subject in order to offer more to the big picture, which is why it is important to try to humanise and unveil science education political nature.

Thus, changing science education means problematising its ethical and political engagement. On the one hand, that problematisation is also important to our ongoing process of the PhD. For instance when questioning the professional development of teacher educators, identity, beliefs, or working conditions that could be influencing the spaces of teacher development such as the CPD (in the case of Paulina); or by questioning the assumptions between the legitimised knowledge that is part of the university with the assumptions of informal/non-legitimated knowledge of the schoolteachers (in the case of Betzabé). Besides, between us, through this dialogue, we also problematised our tensions. We think that we are changing together (considering the differences of our identities) towards a political engagement from what we can reconstruct our practices and a sense of agency in science education. In so doing, in this article, we presented the communities, our sense of agency within science educations and a reread of our history being part of the community.

This feeling of science for social justice and commitment to activism is growing (Tolbert and Bazzul 2016). For us, we should always consider how we can act, how can we open or engage with democratic projects in dialogic solidarity, reshaping the world of science education, and challenging neoliberal ways of relating to each other.

## Footnotes

1 Ian Mitchell is the coordinator of PEEL which is ‘Project for Enhancing Reflective learning’. The original PEEL group was created in 1985.

2 See Acuña et al. (2016) to expand this information or the Master’ thesis of Camilo Henriquez called “Analysis of lesson stories of teachers that reflect for a transforming education in science: structure and changes over time” which explores the structure of the lesson stories as a tool to reflect on teachers’ praxis.
